# Withdrawn medicines included in the essential medicines lists of 136 countries

**DOI:** 10.1371/journal.pone.0225429

**Published:** 2019-12-02

**Authors:** Onella Charles, Igho Onakpoya, Simran Benipal, Hannah Woods, Anjli Bali, Jeffrey K. Aronson, Carl Heneghan, Nav Persaud

**Affiliations:** 1 MAP Centre for Urban Health Solutions, St. Michael’s Hospital, Toronto, Canada; 2 Centre for Evidence-Based Medicine, Nuffield Department of Primary Care Health Sciences, University of Oxford, Oxford, United Kingdom; 3 Department of Family and Community Medicine, St. Michael’s Hospital, Toronto, Ontario; York University, CANADA

## Abstract

**Background:**

Essential medicines lists and related policies are intended to meet the priority health needs of populations and their implementation is associated with more appropriate use of medicines. The World Health Organization (WHO) recommends that countries carefully select the medicines to be included in their national essential medicines lists. Lists that are used to prioritize access to important treatments should not include medicines that have been withdrawn elsewhere because of an unfavourable benefit-to-harm balance; however, countries still list and use medicines that have been withdrawn worldwide. The objective of this study was to determine whether the national essential medicines lists of 137 countries include medicines that have been withdrawn in other countries.

**Methods and findings:**

We performed an audit of national essential medicines lists for medicines that had been withdrawn. Medicines withdrawn from worldwide markets between 1953 and 2014 were identified using a systematic review of published literature and regulatory documents. The reviewers used sources including the WHO’s database of drugs, PubMed, and the websites of regulatory agencies to obtain information regarding adverse effects associated with the medicines, the year of first withdrawal, markets of withdrawal, and the level of evidence supporting each withdrawal. We recorded the number of countries with a withdrawn medicine included in their national medicines list, the number of withdrawn medicines included in each nation’s list, and the number of national essential medicines including each withdrawn medicine. 97 medicines were withdrawn in at least one country but still included in one more national essential medicines list. Of 137 countries with a national essential medicines list, 136 lists included at least one withdrawn medicine, with 54% of the lists containing 5 or fewer withdrawn medicines, and 27% including 10 or more withdrawn medicines. 11 medicines were withdrawn worldwide but still included on at least one national essential medicines list. Countries with longer essential medicines lists had more withdrawn medicines included in their lists.

**Conclusions:**

This study found that withdrawn medicines are included in all but one national essential medicines list, representing a need for more stringent processes for selecting and removing medicines on these lists. Countries may wish to apply special scrutiny to medicines withdrawn in other nations when selecting medicines to include on their lists.

## Introduction

Essential medicines lists and related policies are intended to meet the priority health needs of populations [1]. They can promote health and patient safety and their implementation, especially in low-income countries, is associated with more appropriate use of medicines [2–4]. The World Health Organization (WHO) recommends that countries carefully select the medicines to be included in their national essential medicines lists [5]. Essential medicines lists contain medicines that are publicly paid for and medicines may not be on essential medicines lists but may still be available publicly. Lists that are used to prioritize access to important treatments should not include medicines that have been withdrawn elsewhere because of an unfavourable benefit-to-harm balance.

Delays in the removal of harmful medicines from the market can lead to substantial morbidity and mortality [6]. For example, cyclo-oxygenase 2 (COX2) inhibitors (coxibs) caused approximately 30 000 excess deaths in the United States before they were withdrawn [7, 8]. Sertindole, used in the treatment of schizophrenia, was available in 13 countries until it was withdrawn because of an association with sudden cardiac death [9]. Yet these medicines are still available and used in some countries. The purpose of this study was to determine whether national essential medicines lists include medicines that have been withdrawn in at least one other country. This study includes the 137 countries that have publically posted essential medicines lists. Countries may want to consider removing these withdrawn medicines from their essential medicines lists.

## Methods

### Search Strategy

We identified withdrawn medicines from a systematic review of published literature and regulatory documents, which identified medicines that were withdrawn from markets around the world between 1953 and 2014 because of adverse drug reactions [10]. The reviewers obtained information regarding the year of first withdrawal, the markets of withdrawal, and the time of the first reported adverse drug reactions, using the following sources: the WHO’s database of drugs, Google Scholar, PubMed, textbooks, and the websites of regulatory agencies. The systematic review assessed the evidence supporting each medicine’s withdrawal by documenting the highest level of evidence available before the medicine’s year of first withdrawal. The evidence was classified using the five levels of the Oxford Centre for Evidence Based Medicine: 1 –systematic review; 2 –randomized clinical trials; 3 –non-randomized, cohort, or follow up studies; 4 –case series; 5 –mechanism based reasoning, where lower numbers indicate better evidence [10, 11] [see S2 Table].

We defined a medicine as “a product that is intended to be taken by or administered to a person or animal for one or more of the following reasons: (i) as a placebo; (ii) to prevent a disease; (iii) to make a diagnosis; (iv) to test for the possibility of an adverse effect; (v) to modify a physiological, biochemical, or anatomical function or abnormality; (vi) to replace a missing factor; (vii) to ameliorate a symptom; (viii) to treat a disease; and/or (ix) to induce anesthesia.” [12]

All medicines’ names were recorded as they were listed in the database of national essential medicines lists. We treated bases and their salts as the same medicinal product (e.g. bendazac lysine and bendazac).

In order to determine whether withdrawn medicines were included in a national essential medicines list, we searched a database of the most recent national essential medicines lists derived from lists compiled by the World Health Organization [13].

### Inclusion/exclusion criteria

We included all withdrawn medicines that met one of the following criteria:

- a medicine whose importation, sale, marketing, use, manufacturing, and/or distribution has been prohibited or banned in at least one national market because of adverse effects or reactions or lack of proven efficacy;
- a medicine whose marketing authorization has been suspended, removed, withdrawn, and/or cancelled in at least one market because of adverse effects or reactions or lack of proven efficacy;
- a medicine whose registration has been cancelled, suspended, or refused in at least one national market because of adverse effects or reactions or lack of proven efficacy;
- a medicine whose approval has been withdrawn in at least one national market because of adverse effects or reactions or lack of proven efficacy;
- a medicine whose suspension of market authorization or market withdrawal has been required by a National Regulatory Health Agency because of adverse effects or reactions.

We use the term “withdrawn” to refer to both medicines that have been withdrawn by a regulator after marketing and those that were not approved for marketing because of adverse effects. In the latter case, a regulator has decided that the medicine should not be made available even though the medicine may be available in other markets.

Additionally, all withdrawn medicines that met one of the following criteria were excluded:

- any medicine that was temporarily removed, withdrawn, or suspended, but later reintroduced into the market of withdrawal;
- any medicine whose use is withdrawn, prohibited, or banned only for certain indications (e.g. contraindicated for paediatric use) or any medicine whose use is strictly regulated or controlled (e.g. included in a controlled substance schedule);
- any medicine whose use is withdrawn, prohibited, or banned only for specific formulations, preparations, or routes of administration (e.g. topical or intravenous);
- any medicine that has been withdrawn in one or more national markets but is on the WHO’s Essential Medicines List (EML).

### Withdrawal details

We obtained the years and markets of withdrawal for each medicine by searching the WHO’s consolidated list of products whose consumption or sale have been banned, withdrawn, severely restricted, or not approved by governments (12th and 14th editions) [14, 15]. When medicines were not found in the WHO consolidated list, the following alternative sources were searched: the Drug Bank database [16], websites of national regulatory agencies, including the US Food and Drug Administration and the European Medicines Agency, and Medline. Only markets that withdrew, banned, or prohibited a medicine in all preparations and formulations were considered markets of complete withdrawal.

We obtained information about the adverse drug reactions associated with each medicine from the 2016 systematic review [10]. We also searched Medline for additional information about the adverse drug reactions. The type of evidence supporting each withdrawal was classified using the Oxford Centre for Evidence Based Medicine criteria [10, 11].

### Classification of withdrawal

We classified the reason for withdrawal of each medicine into one of three categories: harm that is unique to the medicine, harm that is general to the chemical subgroup, or lack of proven efficacy [see S2 Table]. This was done by comparing each withdrawn medicine to other medicines in the same therapeutic subgroup. The therapeutic subgroup of each medicine was identified using level 2 of the medicine’s Anatomical Therapeutic Classification (ATC) code [17]. If the adverse effects of the withdrawn medicine were not common among other medicines of the same subgroup, the harm was considered “unique to the medicine” whereas, if similar adverse effects were identified across multiple drugs of the same subgroup, the harm was considered “general to the chemical subgroup”. One reviewer (OC) extracted the data for all 97 withdrawn medicines, and a second reviewer (SB) independently extracted the data for five randomly selected medicines to verify the accuracy of data extraction. Two reviewers (OC and SB) independently assessed the reasons for withdrawal and a third (NP) arbitrated when there were disagreements.

### Patient and public involvement

This audit of essential medicines lists for withdrawn medicines did not directly involve patients or members of the public.

### Data sharing

The datasets analyzed during the current study are available at essentialmeds.org. To access the datasets, click on Global Essential Medicines, and then click “go to website."

## Results

**Fig 1:**
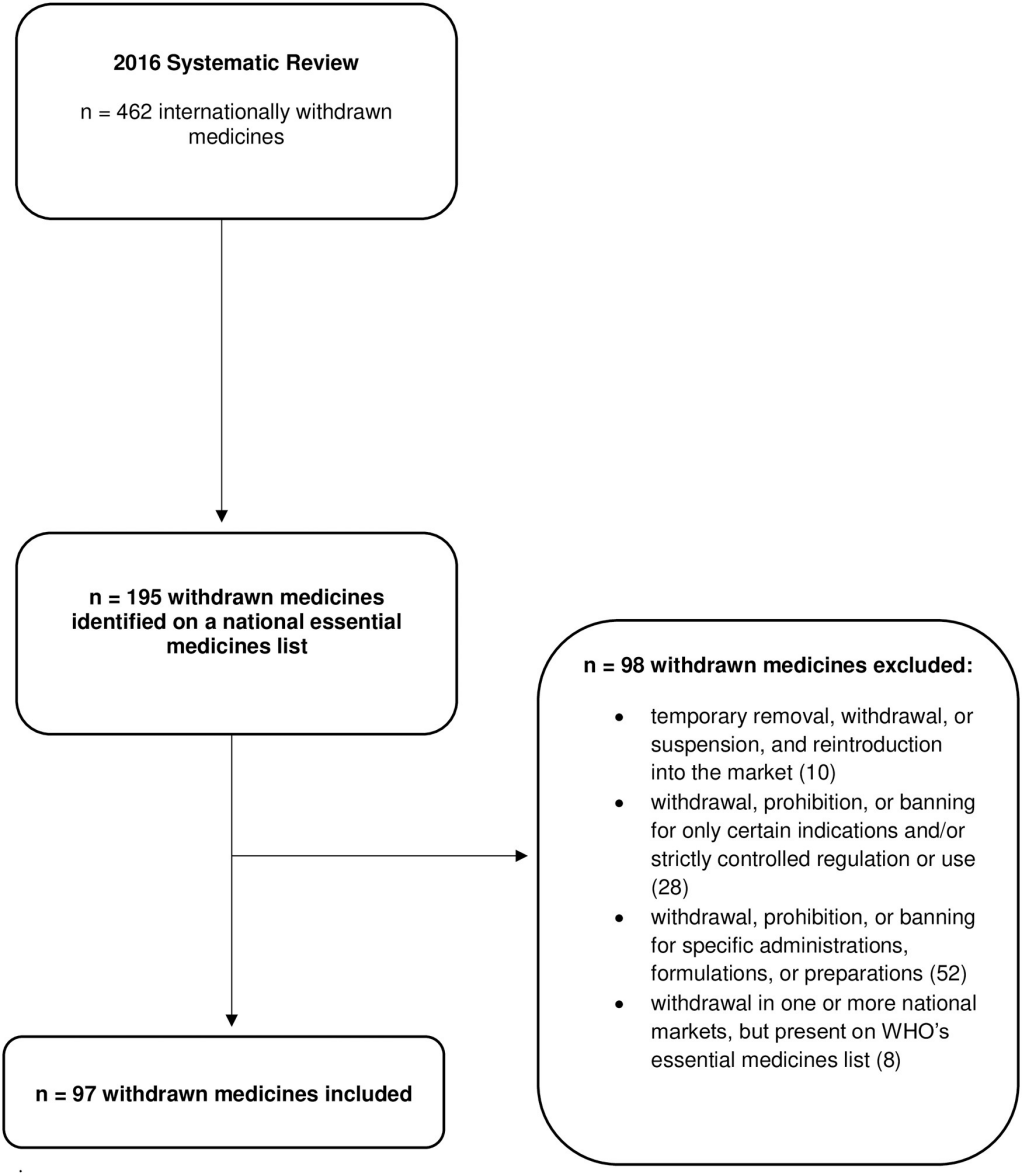
Diagram illustrating inclusion of withdrawn medicines identified on an essential medicines list. The figure shows the process of determining the total number of withdrawn medicines included in this study.

Of the previously identified 462 withdrawn medicines, 195 (42%) were listed on at least one national essential medicines list. After exclusions, we included 97 (21%) withdrawn medicines that were included on at least one national essential medicines list (Fig 1). Eight medicines (buprenorphine, codeine, levamisole, phenobarbital, podophyllum resin, two salts of erythromycin and rotavirus vaccine) were excluded because they were included in the World Health Organization’s model essential medicines list and would thus be included in many national essential medicines lists based on guidance from the World Health Organization. We included rofecoxib as regulators issues statements about safety concerns around the time it was voluntarily withdrawn by the manufacturer.[18, 19] Characteristics of the 137 countries with a national essential medicines list are presented in S1 Table.

Of the 97 withdrawn medicines, the two most common therapeutic subgroups were “psychoanaleptics” (ATC section N06, including antidepressants and psychostimulants; 10%) and “antiinflammatory and antirheumatic products” (ATC section M01; 10%) [see S2 Table]. Of the 97 withdrawn medicines, 73% were withdrawn because of harm unique to the medicine, 25% were withdrawn because of harm general to the chemical subgroup, and 2% were withdrawn because of lack of proven efficacy.

**Fig 2:**
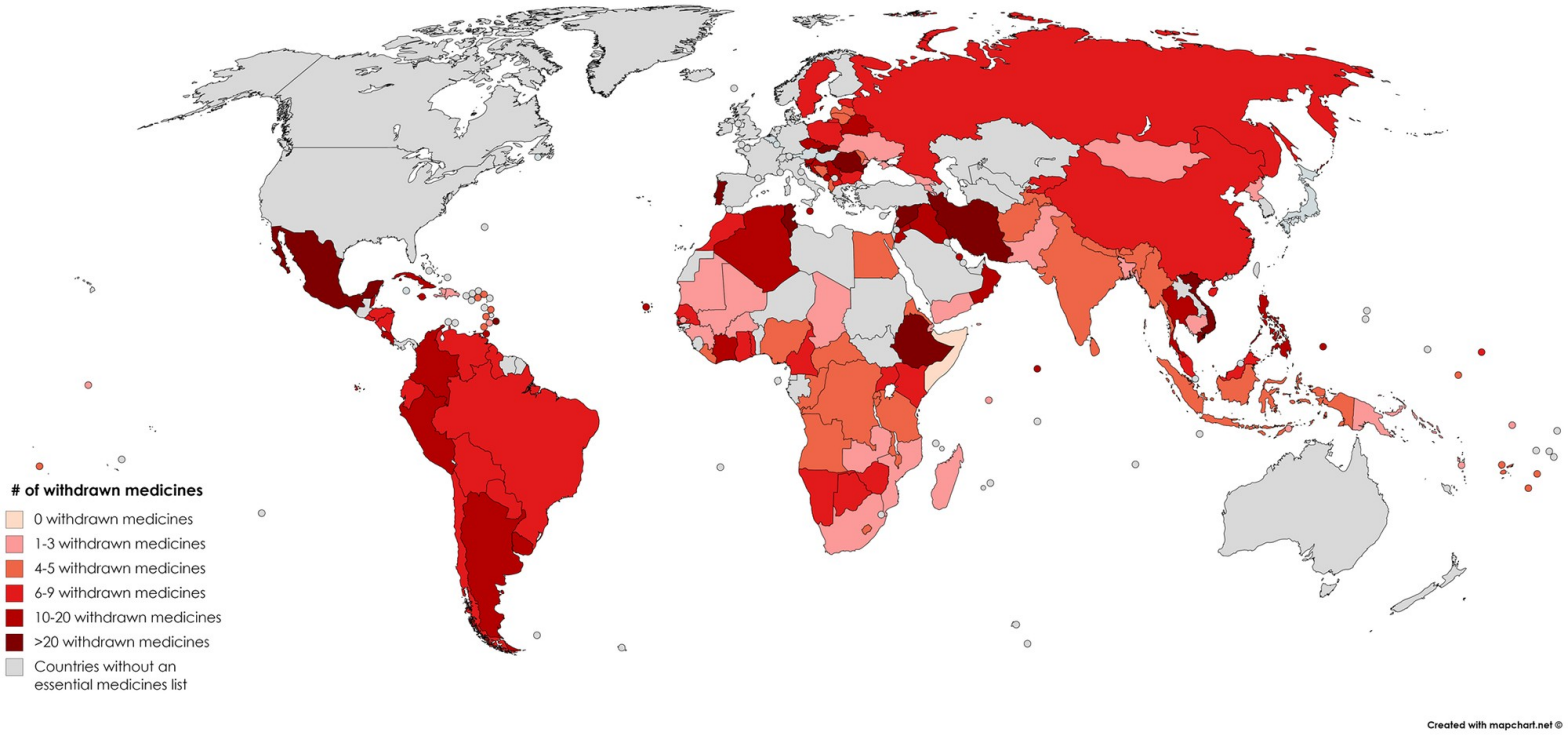
Number of withdrawn medicines included in the national essential medicines lists of 137 countries. The countries that are shaded grey do not have a publically available essential medicines list. Countries are shaded on a color scale where lighter list fewer withdrawn medicines on their essential medicines lists and darker list more.

Most of the medicines (63%) were withdrawn on the basis of case reports (Level 4 evidence). Of the 137 national essential medicines list, 136 included at least one withdrawn medicine (Fig 2).

**Fig 3:**
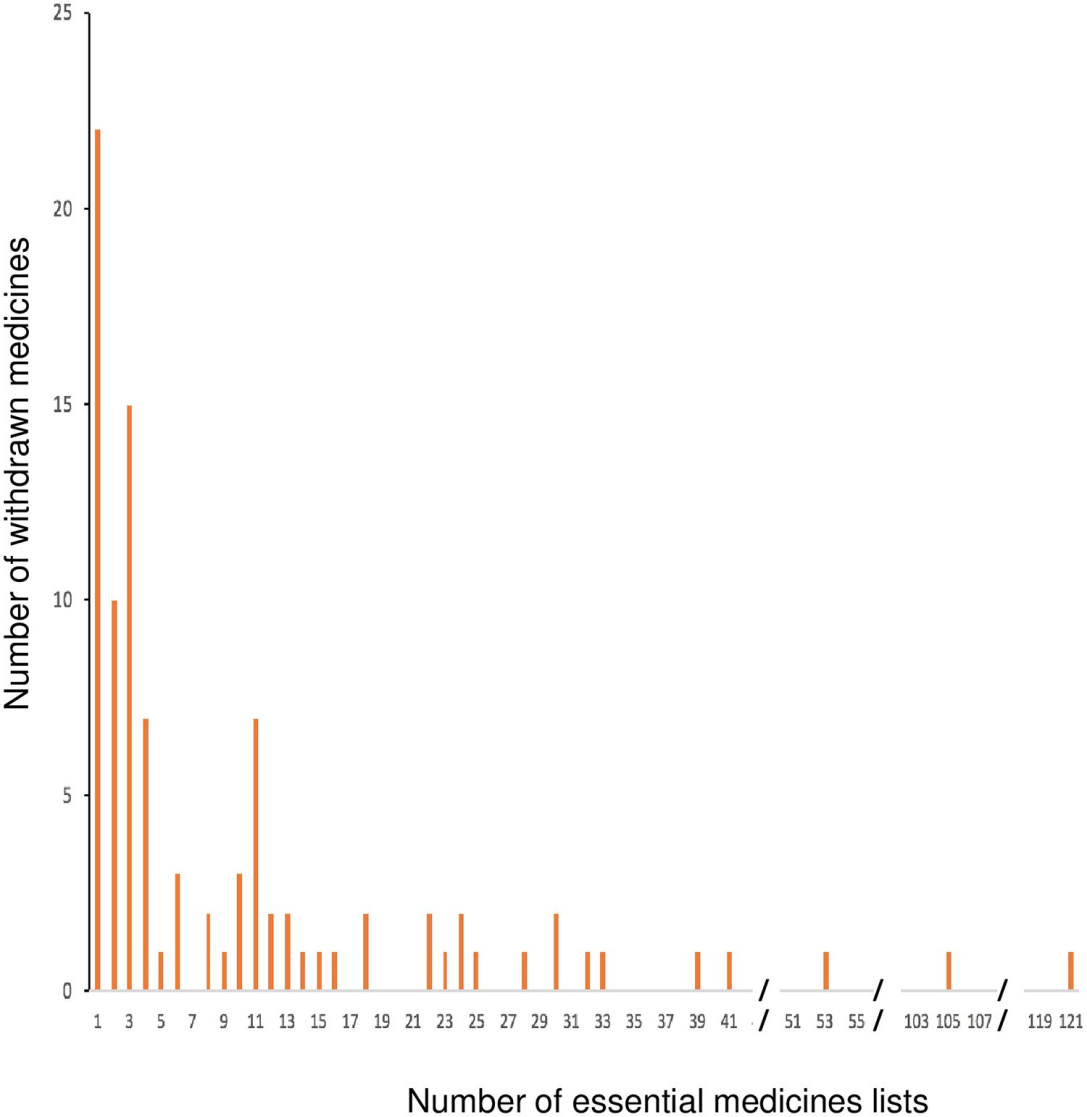
Number of essential medicines lists including withdrawn medicines. The left side of the Figure shows the number of withdrawn medicines included in a small number of national essential medicines lists (e.g. 22 withdrawn medicines are listed by only one national essential medicines list). The right side of the Figure shows withdrawn medicines listed by many countries (e.g. 1 withdrawn medicine is listed by 121 countries).

**Fig 4:**
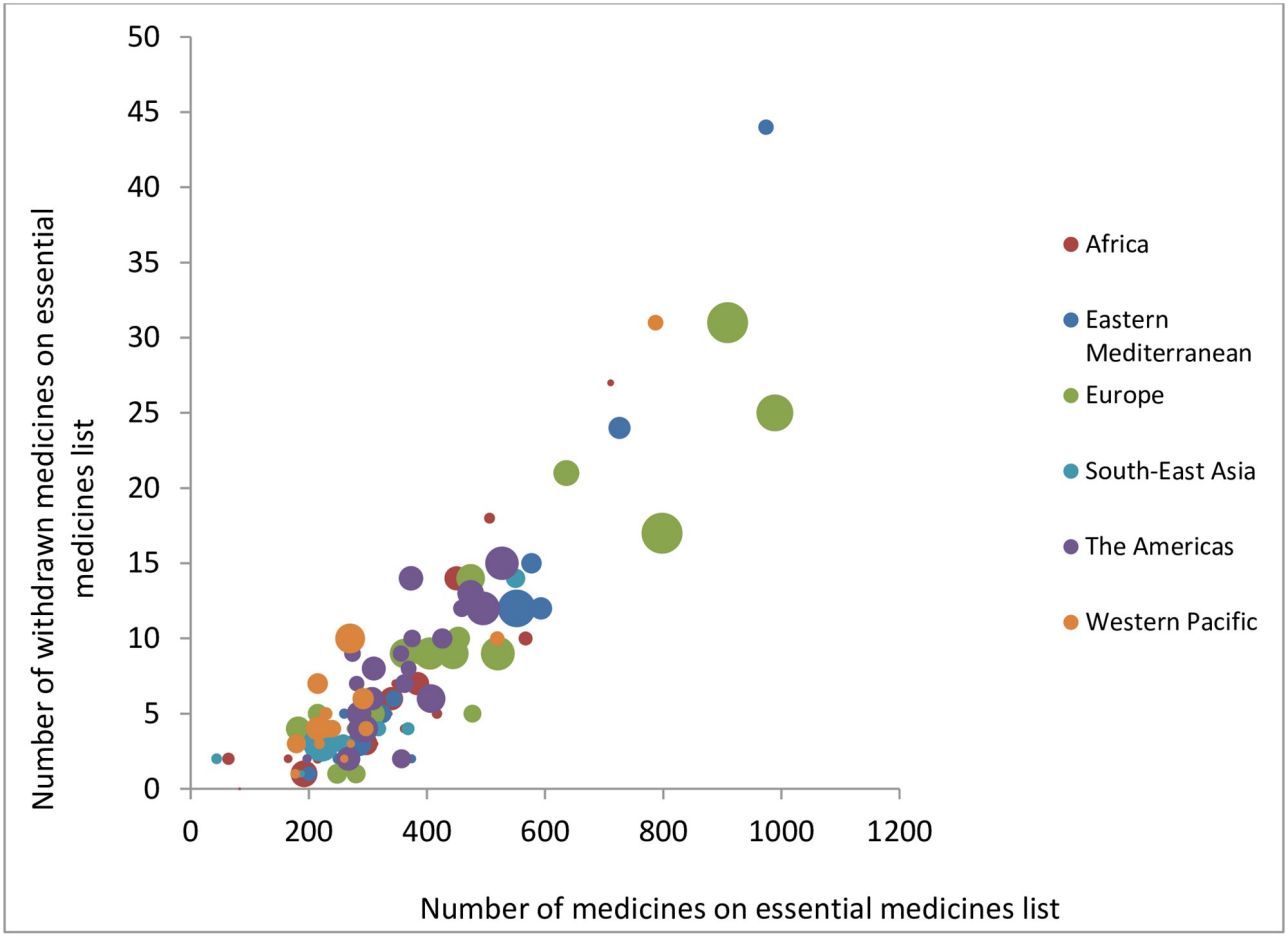
Number of withdrawn medicines included on national essential medicines lists according to the number of medicines on each essential medicines lists, country sub-region, and national GDP. The area of the circle represents national GDP (larger circle, larger national GDP), and the color of the circle represents sub-region.

The number of withdrawn medicines in the 136 countries’ lists ranged from 1 to 44 (median 5, IQR 3–10); 0.4% to 4.5% of the medicines on country lists were withdrawn (Fig 3). Of these 136 countries, 54% listed up to 5 withdrawn medicines, while more than a quarter (27%) listed between 10 and 44. Countries with longer essential medicines lists tended to include more withdrawn medicines (Fig 4).

The number of countries that included each withdrawn medicine on their essential medicines lists ranged from 1 to 121 (median 4, IQR 2–12). Of the 97 withdrawn medicines included, 22 were on one country’s essential medicines list, 42 were listed on 2 to 10 national essential medicines lists, and the remaining 32 were listed on up to 121 national medicines lists.

Of the 97 withdrawn medicines, 11 were withdrawn worldwide (Table 1), 50 were withdrawn only in one or more European countries, 6 were withdrawn only in one or more North American countries, 11 were withdrawn in both Europe and North America, and 19 were withdrawn only in other countries. Seventeen medicines were withdrawn in at least one African country, 45 in at least one Asian country, 9 in at least one Australasian country, and 26 in at least one Central American or South American country.

**Table 1:** Medicines withdrawn worldwide that are included in one or more national essential medicines lists.

Withdrawn medicine	Year of withdrawal	Safety concern	National essential medicines lists including withdrawn medicine and year of most recent list update
Astemizole	1999	Cardiotoxicity, drug-drug interactions	Slovakia—2012 Syrian Arab Republic—2008
Chlormezanone	1996	Toxic epidermal necrosis	Côte d’Ivoire—2014 Ethiopia—2014 Syrian Arab Republic—2008
Drotrecogin alfa	2011	Failure to show benefits	Mexico—2011 Oman—2009 Syrian Arab Republic—2008 The former Yugoslav Republic of Macedonia—2008
Fenfluramine	1997	Cardiovascular, pulmonary	Lesotho—2005
Laropiprant	2013	Increased serious, but nonfatal adverse effects	Portugal—2011
Nebacumab	1993	Accelerated deaths	Bahrain—2015
Nikethamide	1988	Neurotoxicity	China—2012 Cuba—2012 Myanmar—2010
Rofecoxib	2004	Increased risk of heart attacks	Syrian Arab Republic—2008
Suprofen	1986	Nephrotoxicity	Ethiopia—2014
Terodiline	1992	Cardiac arrhythmias	Iraq—2010 Slovenia—2012
Thioridazine	2005	Cardiac arrhythmias, QT prolongation	Antigua and Barbuda—2007 Barbados—2011 Belize—2008 Bolivia—2011 Botswana—2012 Bulgaria—2011 Chile—2010 Colombia—2011 Cuba—2012 Dominica—2007 Eritrea—2010 Ethiopia—2014 Grenada—2007 Guyana—2010 Iran (Islamic Republic)—2014 Kiribati—2009 Lesotho—2005 Maldives—2009 Marshall Islands—2007 Montenegro—2011 Mozambique—2017 Namibia—2016 Nepal—2016 Nicaragua—2011 Oman—2009 Peru—2012 Russian Federation—2014 Saint Kitts and Nevis—2007 Saint Lucia—2007 Saint Vincent and the Grenadines—2010 Serbia—2010 Thailand—2013 The former Yugoslav Republic of Macedonia—2008 Tonga—2007 Trinidad and Tobago—2010 United Republic of Tanzania—2017 Uruguay—2011 Venezuela (Bolivarian Republic)—2004 Viet Nam—2008

Of the 97 withdrawn medicines, 72 were withdrawn in at least one European country. The number of essential medicines lists that included these 71 medicines ranged from 1 to 121 (median 3, IQR 2–11). Twenty-eight (29%) of the 97 medicines were withdrawn in North America and were included in 1 to 39 essential medicines lists (median 3, IQR 1–4). Eleven medicines were withdrawn worldwide and were included in 1 to 39 essential medicines lists (median 2, IQR 1–3) (Table 1).

## Discussion

Although essential medicines are intended to meet priority health needs, all national essential medicines lists bar one contain a medicine that has been withdrawn elsewhere. Over half of the countries include no more than five withdrawn medicines, but more than a quarter (27%) list 10 or more. Most of the 97 withdrawn medicines were listed in more than one list and most were withdrawn on the basis of case reports suggesting harms; 11 were withdrawn worldwide. Some were withdrawn because of serious adverse reactions with an increased risk of death: thioridazine is included in 39 lists, although it was withdrawn worldwide in 2005, cisapride is included in 9 lists, and rosiglitazone is included in 18 lists. As national essential medicines lists are updated, withdrawn medicines should be flagged for removal.

The number of withdrawn medicines correlates with the total number of medicines on EMLs, with those listing a higher total of medicines listing more withdrawn medicines. This could be because countries that list a larger number of medicines have a less stringent process for developing and maintaining their EMLs, and so withdrawn medicines are left on their lists.

In this study we focused on the inclusion of withdrawn medicines in essential medicines lists, while previous studies have shown that some medicines that have been withdrawn in one country continue to be available in other countries. A systematic review showed that of 95 medicines withdrawn in at least one country because of associated deaths, 16 were still marketed elsewhere [6]. A 2015 study showed that 27 medicines withdrawn internationally were still being used in some countries, while a 2012 commentary reported that only 19% of medicines withdrawn in at least one country were also withdrawn internationally [20, 21].

We have comprehensively searched national essential medicines lists for medicines that have been withdrawn elsewhere. There are some limitations to this study. We may have missed some withdrawn medicines, as the 2016 systematic review did not include information from certain African countries, such as Somalia, owing to armed conflict. The systematic review only included medications withdrawn between 1953 and 2014; more medications may have been withdrawn since and may be listed on national essential medicines lists [10]. Companies may voluntarily withdraw medicines when it is clear that the regulator will act to remove them from the market, and these medicines may not appear in the literature we consulted. The database of national essential medicines lists was based on information provided to the World Health Organization, which may have been out of date or abstracted incorrectly [13].

Even when a medicine is withdrawn in a specific country, there may be a delay before these changes are implemented in clinical practice and the medicine is no longer used. Additionally, it is not clear how withdrawn medicines are being used in the countries that include them in their essential medicines lists. A country’s EML may not be used in practice and so listed medicines may not be used. [22] The fact that a medicine has been withdrawn does not necessarily mean that it should not be listed in essential medicines lists; this may be especially so when the evidence supporting withdrawal is poor or limited. For example, neomycin, which was banned in Bangladesh in 1982, is still included on 105 essential medicines lists and may still be a reasonable medicine to include on these lists. On the other hand, medicines listed by majority of countries may still be inappropriately included on essential medicines lists because of their associations with harm. For example, the nonsteroidal anti-inflammatory drug diclofenac, which was refused for registration in the Philippines and Norway in 1983 and 1987 respectively (and later approved for use for the first time in Norway in 2006 [23]), is listed by almost 90% of countries. However, a 2014 study recommended that diclofenac be removed from all essential medicines lists and have its marketing authorization internationally revoked given its similar cardiovascular risks to rofecoxib, a medicine that has been withdrawn worldwide [24]. Medicines may also be rightly withdrawn based on what is considered poor evidence (level 4 for example), if the safety concern is serious enough. Where safety concerns are serious, it would be unethical to seek to establish higher levels of evidence. We excluded some medicines with potentially fatal adverse effects, such as fenoterol (included in 31 lists), because its approval was revoked for only one indication and its use is permitted for mild and moderate asthma.

The presence of withdrawn medicines in national essential medicines lists represents an opportunity for improvement. When selecting medicines for an essential medicines list and when updating existing lists, countries may want to apply special scrutiny to medicines that have been withdrawn in other countries. Countries with longer essential medicines lists may want to adopt more stringent processes that would exclude medicines withdrawn elsewhere. We acknowledge that in low resource settings this activity may have to compete with more pressing demands on the country’s essential medicines program. Universal guidelines for withdrawing medicines may improve communication between international regulatory agencies and reduce the current confusion caused by conflicting regulatory decisions in different countries, although there may be good reasons for different decisions made by different countries. This communication would be aided by a single public site where countries could record their regulatory withdrawal decisions with reasons for their actions. Countries could now prioritize the 11 medicines that have been withdrawn worldwide for removal from their essential medicines lists. The WHO could provide explicit guidance to countries updating their national essential medicines lists to review its list of withdrawn medicines [14, 15]. Future studies could estimate the use and health effects of withdrawn medicines in the countries that list them and compare this to the resources needed to review lists, especially in low- and middle-income countries. This could help determine which withdrawn medicines should no longer be included on any national essential medicines list.

## Supporting information

S1 Table Characteristics of the 137 countries with a national essential medicines list. (DOCX)

S2 Table Characteristics of medicines withdrawn in at least one country that are included in one or more national essential medicines lists. (DOCX)

S1 File STROBE Checklist. (DOCX)

## Abbreviations

ATC: Anatomical Therapeutic Classification
EML: Essential Medicines List
WHO: World Health Organization
