# Supplementary material for: Withdrawn medicines included in the essential medicines lists of 136 countries
Source: PLoS One. 2019 Dec 2;14(12):e0225429. doi: 10.1371/journal.pone.0225429 (PMC6887519; doi:10.1371/journal.pone.0225429)
Supplement: S1 Table — (DOCX) [file pone.0225429.s001.docx]

S1 Table

| **Country** | **Region, World Health Orgnaization** | **Population** | **Life Expectancy (years)** | **GDP per Capita (PPP $)** | **Total number of medicines on the list** |
| --- | --- | --- | --- | --- | --- |
| Afghanistan | Eastern Mediterranean | 34,124,811 | 51.7 | 1900 | 260 |
| Albania | Europe | 3,047,987 | 78.5 | 12,500 | 215 |
| Algeria | Africa | 40,969,443 | 77 | 15,100 | 450 |
| Antigua and Barbuda | The Americas | 94,731 | 76.7 | 26,300 | 294 |
| Angola | Africa | 29,310,273 | 60.2 | 6,800 | 64 |
| Argentina | The Americas | 44,293,293 | 77.3 | 20,700 | 474 |
| Armenia | Europe | 3,045,191 | 74.9 | 9,100 | 271 |
| Bahrain | Eastern Mediterranean | 1,410,942 | 79 | 51,800 | 552 |
| Bangladesh | South-East Asia | 157,826,578 | 73.4 | 4,200 | 187 |
| Barbados | The Americas | 292,336 | 75.5 | 17,500 | 636 |
| Belarus | Europe | 9,549,747 | 73 | 18,600 | 372 |
| Belize | The Americas | 360,346 | 68.9 | 8,300 | 375 |
| Bhutan | South-East Asia | 758,288 | 70.6 | 8,700 | 294 |
| Bolivia | The Americas | 11,138,234 | 69.5 | 7,500 | 356 |
| Bosnia and Herzegovina | Europe | 3,856,181 | 76.9 | 11,400 | 182 |
| Bostwana | Africa | 2,214,858 | 63.3 | 18,100 | 340 |
| Brazil | The Americas | 207,353,391 | 74 | 15,500 | 407 |
| Bulgaria | Europe | 7,101,510 | 74.7 | 21,600 | 362 |
| Burkina Faso | Africa | 20,107,509 | 55.9 | 1,900 | 274 |
| Burundi | Africa | 11,466,756 | 60.9 | 800 | 295 |
| Cambodia | Western Pacific | 16,204,486 | 64.9 | 4,000 | 44 |
| Cameroon | Africa | 24,994,885 | 59 | 3,400 | 353 |
| Cape Verde | Africa | 560,899 | 72.4 | 6,900 | 567 |
| Central African Republic | Africa | 5,625,118 | 52.8 | 700 | 295 |
| Chad | Africa | 12,075,985 | 50.6 | 2,400 | 241 |
| Chile | The Americas | 17,789,267 | 78.9 | 24,600 | 349 |
| China | Western Pacific | 1,379,302,771 | 75.7 | 17,000 | 292 |
| Colombia | The Americas | 47,698,524 | 75.9 | 14,500 | 373 |
| Congo | Africa | 4,954,674 | 59.8 | 6,700 | 303 |
| Cook Islands | Western Pacific | 9,290 | 76 | 16,700 | 240 |
| Costa Rica | The Americas | 4,930,258 | 78.7 | 17,200 | 389 |
| Côte d'Ivoire | Africa | 24,184,810 | 59 | 3,900 | 506 |
| Croatia | Europe | 4,292,095 | 76.1 | 23,400 | 603 |
| Cuba | The Americas | 11,147,407 | 78.8 | 12,300 | 511 |
| Czech Republic | Europe | 10,674,723 | 78.8 | 35,200 | 806 |
| Democratic People’s Republic of Korea | South-East Asia | 51,181,299 | 82.5 | 39,400 | 221 |
| Democratic Republic of Congo | Africa | 83,301,151 | 57.7 | 800 | 314 |
| Djibouti | Africa | 865,267 | 63.6 | 3,600 | 201 |
| Dominica | The Americas | 73,897 | 77.2 | 12,000 | 285 |
| Dominican Republic | The Americas | 10,734,247 | 78.3 | 17,000 | 357 |
| Ecuador | The Americas | 16,290,913 | 77 | 11,200 | 369 |
| Egypt | Eastern Mediterranean | 97,041,072 | 73 | 13,000 | 324 |
| El Salvador | The Americas | 6,172,011 | 74.9 | 8,900 | 362 |
| Eritrea | Africa | 5,918,919 | 65.2 | 1,400 | 337 |
| Estonia | Europe | 1,251,581 | 76.9 | 31,500 | 405 |
| Ethiopia | Africa | 105,350,020 | 62.6 | 2,100 | 711 |
| Fiji | Western Pacific | 920,938 | 73 | 9,900 | 297 |
| Gambia | Africa | 2,051,363 | 65.1 | 1,700 | 165 |
| Georgia | Europe | 4,926,330 | 76.4 | 10,600 | 248 |
| Ghana | Africa | 27,499,924 | 67 | 4,600 | 303 |
| Grenada | The Americas | 111,724 | 74.5 | 14,700 | 284 |
| Guinea | Africa | 12,413,867 | 61 | 2,000 | 239 |
| Guyana | The Americas | 737,718 | 68.6 | 8,300 | 281 |
| Haiti | The Americas | 10,646,714 | 64.2 | 1,800 | 197 |
| Honduras | The Americas | 9,038,741 | 71.2 | 5,500 | 369 |
| India | South-East Asia | 1,281,935,911 | 68.8 | 7,200 | 368 |
| Indonesia | South-East Asia | 260,580,739 | 73 | 12,400 | 279 |
| Iran (Islamic Republic of) | Eastern Mediterranean | 82,021,564 | 74 | 20,000 | 896 |
| Iraq | Eastern Mediterranean | 39,192,111 | 74.9 | 17,000 | 577 |
| Jamaica | The Americas | 2,990,561 | 73.7 | 9,200 | 459 |
| Jordan | Eastern Mediterranean | 10,248,069 | 74.8 | 12,500 | 593 |
| Kenya | Africa | 47,615,739 | 64.3 | 3,500 | 417 |
| Kiribati | Western Pacific | 108,145 | 66.5 | 1,900 | 218 |
| Kyrgyzstan | Europe | 5,789,122 | 70.9 | 3,700 | 316 |
| Latvia | Europe | 1,944,643 | 74.7 | 27,300 | 308 |
| Lebanon | Eastern Mediterranean | 6,229,794 | 77.8 | 19,500 | 285 |
| Lesotho | Africa | 1,958,042 | 53 | 3,900 | 195 |
| Liberia | Africa | 4,689,021 | 63.3 | 900 | 215 |
| Lithuania | Europe | 2,823,859 | 75 | 31,900 | 340 |
| Madagascar | Africa | 25,054,161 | 66.3 | 1,600 | 253 |
| Malawi | Africa | 19,196,246 | 61.7 | 1,200 | 324 |
| Malaysia | Western Pacific | 31,381,992 | 75.2 | 28,900 | 310 |
| Maldives | South-East Asia | 392,709 | 75.8 | 19,200 | 539 |
| Mali | Africa | 17,885,245 | 60.3 | 2,200 | 285 |
| Malta | Europe | 416,338 | 80.5 | 42,500 | 612 |
| Marshall Islands | Western Pacific | 74,539 | 73.4 | 3,400 | 215 |
| Mauritania | Africa | 3,758,571 | 63.4 | 4,500 | 215 |
| Mexico | The Americas | 124,574,795 | 76.1 | 19,500 | 709 |
| Mongolia | Western Pacific | 3,068,243 | 69.9 | 12,600 | 258 |
| Montenegro | Europe | 642,550 | 76.3 | 17,400 | 453 |
| Morocco | Eastern Mediterranean | 33,986,655 | 77.1 | 8,600 | 344 |
| Mozambique | Africa | 26,573,706 | 53.7 | 1,300 | 259 |
| Myanmar | South-East Asia | 55,123,814 | 68.2 | 6,300 | 315 |
| Namibia | Africa | 2,484,780 | 64 | 11,500 | 384 |
| Nauru | Western Pacific | 11,359 | 67.4 | 12,200 | 231 |
| Nepal | South-East Asia | 29,384,297 | 71 | 2,700 | 300 |
| Nicaragua | The Americas | 6,025,951 | 73.5 | 5,800 | 274 |
| Nigeria | Africa | 190,632,261 | 53.8 | 5,900 | 306 |
| Niue | Western Pacific | 1,618 | N/A | 5,800 | 215 |
| Oman | Eastern Mediterranean | 4,613,241 | 75.7 | 45,500 | 578 |
| Pakistan | Eastern Mediterranean | 204,924,861 | 68.1 | 5,400 | 374 |
| Palau | Western Pacific | 21,431 | 73.4 | 16,700 | 270 |
| Papua New Guinea | Western Pacific | 6,909,701 | 67.3 | 3,800 | 271 |
| Paraguay | The Americas | 6,943,739 | 77.4 | 9,800 | 307 |
| Peru | The America | 31,036,656 | 74 | 13,300 | 426 |
| Philippines | Western Pacific | 104,256,076 | 69.4 | 8,200 | 519 |
| Poland | Europe | 38,476,269 | 77.8 | 29,300 | 444 |
| Portugal | Europe | 10,839,514 | 79.4 | 30,300 | 909 |
| Republic of Moldova | Europe | 3,474,121 | 71 | 5,700 | 477 |
| Romania | Europe | 21,529,967 | 75.4 | 24,000 | 636 |
| Russian Federation | Europe | 142,257,519 | 71 | 27,900 | 520 |
| Rwanda | Africa | 11,901,484 | 64.3 | 2,100 | 288 |
| Saint Kitts and Nevis | The Americas | 52,715 | 75.9 | 26,800 | 291 |
| Saint Lucia | The Americas | 164,994 | 77.9 | 13,500 | 291 |
| Saint Vincent and the Grenadines | The Americas | 102,089 | 75.5 | 11,600 | 267 |
| Senegal | Africa | 14,668,522 | 62.1 | 2,700 | 339 |
| Serbia | Europe | 7,111,024 | 75.7 | 15,200 | 474 |
| Seychelles | Africa | 93,920 | 74.9 | 28,700 | 297 |
| Slovakia | Europe | 5,445,829 | 77.3 | 32,900 | 989 |
| Slovenia | Europe | 1,972,126 | 78.3 | 34,100 | 798 |
| Solomon Islands | Western Pacific | 647,581 | 75.6 | 2,100 | 260 |
| Somalia | Africa | 11,031,386 | 52.8 | 434 |  |
| South Africa | Africa | 54,841,552 | 63.8 | 13,400 | 192 |
| Sri Lanka | South-East Asia | 22,409,381 | 76.9 | 13,000 | 318 |
| Sudan | Eastern Mediterranean | 37,345,935 | 64.4 | 4,600 | 302 |
| Suriname | The Americas | 591,919 | 72.5 | 13,900 | 285 |
| Sweden | Europe | 9,960,487 | 82.1 | 51,300 | 289 |
| Syrian Arab Republic | Eastern Mediterranean | 18,028,549 | 75.1 | 2,900 | 974 |
| Tajikistan | Europe | 8,468,555 | 68.1 | 3,100 | 273 |
| Thailand | South-East Asia | 68,414,135 | 74.9 | 17,800 | 550 |
| The former Yugoslav Republic of Macedonia | Europe | 2,103,721 | 76.4 | 15,200 | 391 |
| Timor-Leste | South-East Asia | 1,291,358 | 68.4 | 5,000 | 240 |
| Togo | Africa | 7,965,055 | 65.4 | 1,600 | 297 |
| Tonga | Western Pacific | 106,479 | 76.4 | 5,600 | 229 |
| Trinidad and Tobago | The Americas | 1,218,208 | 73.1 | 31,200 | 495 |
| Tunisia | Eastern Mediterranean | 11,403,800 | 75.7 | 11,900 | 726 |
| Tuvalu | Western Pacific | 11,052 | 66.9 | 3,800 | 179 |
| Uganda | Africa | 39,570,125 | 55.9 | 2,400 | 365 |
| Ukraine | Europe | 44,033,874 | 72.1 | 8,700 | 280 |
| United Republic of Tanzania | Africa | 53,950,935 | 62.6 | 3,300 | 362 |
| Uruguay | The Americas | 3,360,148 | 77.4 | 22,400 | 527 |
| Vanuatu | Western Pacific | 282,814 | 73.7 | 2,800 | 177 |
| Venezuela (Bolivarian Republic of) | The Americas | 31,304,016 | 76 | 12,400 | 310 |
| Viet Nam | Western Pacific | 96,160,163 | 73.7 | 6,900 | 787 |
| Yemen | Eastern Mediterranean | 28,036,829 | 65.9 | 2,300 | 250 |
| Zambia | Africa | 15,972,000 | 52.7 | 4,000 | 288 |
| Zimbabwe | Africa | 13,805,084 | 60.4 | 2,300 | 347 |
