# Supplementary material for: Withdrawn medicines included in the essential medicines lists of 136 countries
Source: PLoS One. 2019 Dec 2;14(12):e0225429. doi: 10.1371/journal.pone.0225429 (PMC6887519; doi:10.1371/journal.pone.0225429)
Supplement: S2 Table — (DOCX) [file pone.0225429.s002.docx]

S2 Table

| **Withdrawn medicine** | **Therapeutic subgroup (ATC level 2 code)** | **Therapeutic subgroup description** | **Reason for withdrawal** | **Safety concern** | **Evidence for withdrawal (levels 1-5)** | **Number of lists that include the withdrawn medicine N=137** | **Number of markets that have withdrawn the medicine** | **Markets of withdrawal** | **Year of first withdrawal** |
| --- | --- | --- | --- | --- | --- | --- | --- | --- | --- |
| Amineptine | N06 | Psychoanaleptics | Harm unique to the medicine | Abuse, hepatotoxicity, skin reactions | 4 | 1 | 5 | France, Thailand, United Arab Emirates, Morocco, Viet Nam | 1999 |
| Astemizole | R06 | Antihistamines for systemic use | Harm general to the chemical subgroup | Cardiotoxicity, drug-drug interactions | 4 | 2 | 14 | Worldwide; Norway, Philippines, USA, South Africa, Canada, United Arab Emirates, Mauritius, Brunei, Tanzania, Armenia, Brazil, Argentina, Singapore | 1987 |
| Bendazac lysine | M02 | Topical products for joint and muscular pain | Harm unique to the medicine | Hepatotoxicity | 4 | 2 | 1 | Spain | 1993 |
| Benfluorex | A10 | Drugs used in diabetes | Harm unique to the medicine | Cardiotoxicity, deaths | 3 | 1 | 1 | Europe | 2009 |
| Benzbromarone | M04 | Antigout preparations | Harm unique to the medicine | Hepatic damage | 4 | 3 | 2 | France, Portugal | 2003 |
| Bismuth | A02 | Drugs for acid related disorders | Harm unique to the medicine | Encephalopathy | 4 | 41 | 3 | Bangladesh, Mauritius, India | 1982 |
| Bromfenac | S01 | Ophthalmologicals | Harm unique to the medicine | Liver failure | 4 | 1 | 2 | USA, Saudia Arabia | 1998 |
| Buflomedil | C04 | Peripheral vasodilators | Harm unique to the medicine | Neurotoxicity, cardiotoxicity | 4 | 4 | 1 | EU | 2011 |
| Buformin | A10 | Drugs used in diabetes | Harm general to the chemical subgroup | Lactic acidosis | 4 | 1 | 4 | Germany, Austria, Belgium, Ireland | 1978 |
| Carisoprodol | M03 | Muscle relaxants | Harm unique to medicine | Abuse potential | 4 | 2 | 3 | EU, Sweden, Norway | 2007 |
| Chenodeoxycholic acid | A05 | Bile and liver therapy | Harm unique to the medicine | Tumorigenicity | 5 | 3 | 1 | Norway | 1987 |
| Chloral hydrate | N05 | Psycholeptics | Harm unique to the medicine | Tumorigenicity | 5 | 18 | 3 | France, India, USA | 2001 |
| Chlormadinone | G03 | Sex hormones and modulators of the genital system | Harm unique to the medicine | Tumorigenicity | 5 | 8 | 3 | Italy, Egypt, Venezuela | 1979 |
| Chlormezanone | M03 | Muscle relaxants | Harm unique to the medicine | Toxic epidermal necrosis | 4 | 3 | 6 | Worldwide; United Arab Emirates, South Africa, Zimbabwe, Saudi Arabia, Singapore | 1996 |
| Chloroform | N01 | Anesthetics | Harm unique to the medicine | Cardiotoxicity, tumorigenicity | 5 | 3 | 18 | Greece, Japan, USA, Panama, Saudi Arabia, Brazil, Italy, Canada, Norway, Philippines, Denmark, Ethiopia, Germany, Bangladesh, Belgium, Nigeria, Ireland, Oman | 1976 |
| Cisapride | A03 | Drugs for functional gastrointestinal disorders | Harm unique to the medicine | Cardiac arrhythmias | 4 | 9 | 14 | Philippines, Oman, USA, Germany, UK, Mauritius, Canada, Brunei, Turkey, Japan, Signapore, Cuba, Bahrain, Armenia | 2000 |
| Clioquinol | D08 | Antiseptics and disinfectants | Harm general to the chemical subgroup | Subacute myelo-optic neuropathy (SMON), neurotoxicity | 4 | 11 | 19 | Japan, Norway, Denmark, United Arab Emirates, Nigeria, Bangladesh, Philippines, Italy, Nepal, Dominican Republic, Zimbabwe, Spain, Hong Kong, Ethiopia, Honduras, Ghana, Bahrain, Netherlands, Saudi Arabia | 1970 |
| Clobutinol | R05 | Cough and cold preparations | Harm unique to the medicine | Long QT syndrome, cardiac arrhythmias | 4 | 5 | 2 | EU, Argentina | 2007 |
| Clofibrate | C10 | Lipid modifying agents | Harm general to chemical subgroup | Accelerated deaths, ventricular arrhythmias | 4 | 3 | 3 | Israel, Norway, Bangladesh | 1979 |
| Cobalt chloride | N/A | N/A | Harm unique to the medicine | Cardiotoxicity | 4 | 2 | 2 | USA, Kuwait | 1967 |
| Dibenzepin | N06 | Psychoanaleptics | Harm unique to the medicine | Suicide | 4 | 2 | 1 | Sweden | 1983 |
| Diclofenac | M01 | Antiinflammatory and antirheumatic products | Harm unique to the medicine | Gastrointestinal, skin reactions | 4 | 121 | 2 | Philippines, Norway | 1983 |
| Dienestrol | G03 | Sex hormones and modulators of the genital system | Harm general to the chemical subgroup | Carcinogenicity | 3 | 4 | 2 | Italy, Kuwait | 1979 |
| Diethylstilbestrol | G03 | Sex hormones and modulators of the genital system | Harm general to the chemical subgroup | Tumorigenicity | 3 | 11 | 4 | Panama, Austria, Kuwait, Italy | 1973 |
| Droperidol (Dehydrobenoperidol) | N05 | Psycholeptics | Harm unique to the medicine | Cardiotoxicity | 4 | 33 | 2 | UK, Indonesia | 2001 |
| Drotrecogin alfa | B01 | Antithrombotic agents | Lack of proven efficacy | Failure to show benefits | 1 | 4 | 1 | Worldwide | 2011 |
| Emetine | P01 | Antiprotozoals | Harm unique to the medicine | Cardiotoxicity | 4 | 1 | 1 | Mauritius | 1982 |
| Etretinate | D05 | Antipsoriatics | Harm general to the chemical subgroup | Teratogenicity | 5 | 3 | 2 | Norway, Brazil | 1992 |
| Fenfluramine | A08 | Antiobesity preparations, excluding diet products | Harm unique to the medicine | Cardiovascular, pulmonary | 3 | 1 | 3 | Worldwide; United Arab Emirates, Philippines | 1997 |
| Fluvoxamine | N06 | Psychoanaleptics | Harm unique to the medicine | Teratogenicity, nephrotoxicity | 5 | 22 | 1 | Iceland | 1987 |
| Furazolidone | G01 | Gynaecological antiinfectives and antiseptics | Harm unique to the medicine | Carcinogenic, skin | 4 | 13 | 5 | Japan, Iraq, Malaysia, Republic of Korea, Yemen | 1977 |
| Gatifloxacin | J01 | Antibacterials for systemic use | Harm unique to the medicine | Dysglycemia | 3 | 10 | 4 | Malaysia, Canada, USA, India | 2006 |
| Gemfibrozil | C10 | Lipid modifying agents | Harm general to the chemical subgroup | Adverse effects not balanced by benefits | 2 | 30 | 1 | Norway | 1987 |
| Herpes Virus Vaccine | J07 | Vaccines | Harm unique to the medicine | Potential harmfulness | 5 | 1 | 3 | Germany, Saudi Arabia, Venezuela | 1984 |
| Hexachlorophene | D08 | Antiseptics and disinfectants | Harm unique to the medicine | Mutagenicity, teratogenicity | 3 | 3 | 1 | Russia | 1988 |
| Isocarboxazid | N06 | Psychoanaleptics | Harm general to the chemical subgroup | Metabolic, drug-drug interactions | 4 | 1 | 3 | Japan, Cuba, Venezuela | 1974 |
| Kaolin (light BP powder) | A07 | Antidiarrheals, intestinal anti-inflammatory/ antiinfective gents | Lack of proven efficacy | No evidence it works for its purpose | 4 | 4 | 2 | India, Sri Lanka | 1991 |
| Ketorolac | M01 | Antiinflammatory and antirheumatic products | Harm unique to the medicine | Gastrointestinal, skin reactions | 4 | 30 | 3 | Germany, France, Jamaica | 1993 |
| Laropiprant | N/A | N/A | Harm unique to the medicine | Increased serious, but nonfatal adverse effects | 2 | 1 | 1 | Worldwide | 2013 |
| Lindane (gamma benzene hexachloride) | P03 | Ectoparasiticides, incl. scabicides, insecticides and repellents | Harm unique to the medicine | Potential toxicity | 4 | 24 | 1 | Brazil | 2001 |
| Loxoprofen | M01 | Antiinflammatory and antirheumatic products | Harm unique to the medicine | Colonic ulceration | 4 | 1 | 1 | Singapore | 2000 |
| Lumiracoxib | M01 | Antiinflammatory and antirheumatic products | Harm general to chemical subgroup | Liver failure | 4 | 1 | 8 | New Zealand, Australia, Canada, UK, European Agency, Brazil, Philippines, Colombia | 2007 |
| Meclizine | R06 | Antihistamines for systemic use | Harm unique to the medicine | Teratogenic potential | 5 | 16 | 1 | Indonesia | 1963 |
| Megestrol | G03 | Sex hormones and modulators of the genital system | Harm unique to the medicine | Tumorigenicity | 5 | 28 | 1 | New Zealand | 1976 |
| Mephenesin | M03 | Muscle relaxants | Harm unique to the medicine | Cardiotoxicity | 4 | 3 | 2 | Japan, Saudi Arabia | 1976 |
| Meprobamate | N05 | Psycholeptics | Harm unique to the medicine | Abuse | 4 | 10 | 1 | Canada | 2013 |
| Metamizole | N02 | Analgesics | Harm general to the chemical subgroup | Agranulocytosis | 4 | 34 | 20 | Australia, Norway, USA, Denmark, Saudi Arabia, United Arab Emirates, Malaysia, Ghana, Sri Lanka, Nepal, Syria, Yemen, Zimbabwe, Sweden, Morocco, Armenia, Bahrain, Ireland, Singapore, Venezuela | 1965 |
| Metaproterenol (Orciprenaline) | R03 | Drugs for obstructive airway diseases | Harm unique to the medicine | Cardiotoxicity | 4 | 6 | 1 | UK | 2009 |
| Methylphenidate | N06 | Psychoanaleptics | Harm general to the chemical subgroup | Abuse | 4 | 53 | 3 | Turkey, Oman, Nigeria | 1982 |
| Mianserine | N06 | Psychoanaleptics | Harm unique to the medicine | Agranulocytosis | 4 | 13 | 1 | Oman | 1986 |
| Minocycline | A01 | Stomatological preparations | Harm unique to the medicine | Dizziness, vertigo | 4 | 14 | 1 | Norway | 1989 |
| Nebacumab | J06 | Immune sera and immunoglobulins | Harm unique to the medicine | Accelerated deaths | 2 | 1 | 1 | Worldwide | 1993 |
| Neomycin | A01 | Stomatological preparations | Harm unique to the medicine | Abuse | 4 | 105 | 1 | Bangladesh | 1982 |
| Nifuroxazide | N07 | Other nervous system drugs | Harm unique to the medicine | Immune and hematological toxicity | 4 | 11 | 1 | Belgium | 2008 |
| Nikethamide | R07 | Other respiratory system products | Harm unique to the medicine | Neurotoxicity | 4 | 3 | 1 | Worldwide | 1988 |
| Nimesulide | M01 | Antiinflammatory and antirheumatic products | Harm unique to the medicine | Hepatotoxicity | 4 | 12 | 3 | Nigeria, Ireland, Ghana | 2005 |
| Nitroxoline | J01 | Antibacterials for systemic use | Harm unique to the medicine | Cataracts | 5 | 6 | 3 | Ireland, Thailand, Venezuela | 1973 |
| Parecoxib | M01 | Antiinflammatory and antirheumatic products | Harm general to the chemical subgroup | Cardiotoxicity | 2 | 4 | 1 | USA | 2005 |
| Pergolide mesylate | N04 | Anti-Parkinson drugs | Harm unique to the medicine | Cardiac valve damage | 3 | 4 | 2 | USA, Canada | 2007 |
| Phenazone | N02 | Analgesics | Harm unique to the medicine | Agranulocytosis, aplastic anaemia | 4 | 11 | 3 | United Arab Emirates, Malaysia, Bahrain | 1981 |
| Phenazopyridine | G04 | Urologicals | Harm unique to the medicine | Carcinogenicity | 5 | 11 | 1 | Greece | 1984 |
| Phentermine | A08 | Antiobesity preparations excl. diet products | Harm unique to the medicine | Abuse | 4 | 1 | 5 | United Arab Emirates, Turkey, Oman, UK, Venezuela | 1981 |
| Phentolamine | C04 | Diuretics | Harm unique to the medicine | Carcinogenicity | 5 | 12 | 1 | Singapore | 2000 |
| Phenylbutazone | M01 | Antiinflammatory and antirheumatic products | Harm unique to the medicine | Aplastic anemia, agranulocytosis | 4 | 1 | 9 | United Arab Emirates, Jordan, Ethiopia, Chile, Malaysia, Ghana, Sri Lanka, Armenia, Bahrain | 1984 |
| Phenylpropanolamine | R01 | Nasal preparations | Harm unique to the medicine | Haemorrhagic stroke | 4 | 3 | 12 | Germany, Brazil, Malaysia, Singapore, USA, Oman, Canada, Cuba, Cameroon, Nigeria, Portugal, Timor-Leste | 1987 |
| Phthalylsulfathiazole | A07 | Antidiarrheals, intestinal anti-inflammatory/ antiinfective gents | Harm unique to the medicine | Granulocytopenia | 4 | 1 | 1 | Bangladesh | 1982 |
| Pioglitazone | A10 | Drugs used in diabetes | Harm unique to the medicine | Risk of bladder cancer | 3 | 22 | 1 | France | 2011 |
| Practolol | C07 | Beta blocking agents | Harm unique to the medicine | Skin reactions, neurotoxicity, gastrointestinal | 4 | 1 | 10 | Greece, Turkey, New Zealand, Thailand, Singapore, Mauritius, India, Germany, Norway, Venezuela | 1975 |
| Propyphenazone | N02 | Analgesics | Harm general to the chemical subgroup | Blood dyscrasias | 4 | 3 | 4 | Turkey, United Arab Emirates, Bahrain, Ireland | 1986 |
| Pseudoephedrine | R01 | Nasal preparations | Harm unique to the medicine | Neurotoxicity, gastrointestinal | 3 | 24 | 2 | Colombia, Thailand | 2008 |
| Pyritinol | N06 | Psychoanaleptics | Harm unique to the medicine | Risk of misuse, insufficient therapeutic value | 4 | 8 | 1 | Bangladesh | 1982 |
| Rimonabant | A08 | Antiobesity preparations excl. diet products | Harm unique to the medicine | Psychiatric | 1 | 1 | 2 | Europe, India | 2007 |
| Rofecoxib | M01 | Antiinflammatory and antirheumatic products | Harm general to the chemical subgroup | Increased risk of heart attacks | 1 | 1 | 1 | Worldwide | 2004 |
| Rosiglitazone | A10 | Drugs used in diabetes | Harm unique to the medicine | Cardiotoxicity | 1 | 18 | 5 | New Zealand, South Africa, UK, India, Spain | 2011 |
| Sertindole | N05 | Psycholeptics | Harm unique to the medicine | Cardiotoxicity | 2 | 11 | 2 | UK, Bulgaria | 1998 |
| Sibutramine | A08 | Antiobesity preparations excl. diet products | Harm unique to the medicine | Cardiotoxicity | 4 | 2 | 10 | Australia, Canada, Honk Kong, India, Mexico, New Zealand, Philippines, Thailand, UK, EU | 2002 |
| Sulfacetamide | S01 | Ophthalmologicals | Harm general to chemical subgroup | Eye, skin reactions | 4 | 23 | 1 | Germany | 1992 |
| Sulfadimidine | J01 | Antibacterials for systemic use | Harm general to chemical subgroup | Haematologic | 4 | 1 | 1 | Germany | 1992 |
| Sulfaguanidine | A07 | Antidiarrheals, intestinal anti-inflammatory/ antiinfective gents | Harm general to chemical subgroup | Haematologic | 4 | 3 | 7 | Dominican Republic, Iran, Turkey, Germany, Armenia, Denmark, Venezuela | 1971 |
| Sulfanilamide | J01 | Antibacterials for systemic use | Harm general to chemical subgroup | Immunologic | 4 | 2 | 1 | Germany | 1992 |
| Sulfathiazole | J01 | Antibacterials for systemic use | Harm general to chemical subgroup | Nephrotoxicity, hepatotoxicity, skin reactions | 4 | 3 | 3 | USA, Dominican Republic, France | 1970 |
| Suprofen | M01 | Antiinflammatory and antirheumatic products | Harm unique to the medicine | Nephrotoxicity | 4 | 1 | 1 | Worldwide | 1986 |
| Tegaserod maleate | A06 | Drugs used for constipation | Harm unique to the medicine | Increased risk of heart attacks and strokes | 1 | 2 | 5 | Jordan, Australia, Switzerland, China, Argentina | 2007 |
| Terfenadine | R06 | Antihistamines for systemic use | Harm general to the chemical subgroup | Cardiotoxicity | 4 | 3 | 13 | Oman, France, Morocco, USA, Mauritius, France, Iceland, Saudi Arabia, Brazil, Chile, Argentina, Singapore, Canada | 1997 |
| Terodiline | G04 | Urologicals | Harm unique to the medicine | Cardiac arrhythmias | 4 | 2 | 1 | Worldwide | 1992 |
| Tetrazepam | M03 | Muscle relaxants | Harm unique to the medicine | Skin reactions | 4 | 11 | 1 | EU | 2013 |
| Thalidomide | L04 | Immunosuppressants | Harm unique to the medicine | Teratogenicity | 4 | 25 | 8 | Belgium, Finland, Indonesia, Brazil, Denmark, India, Singapore, Venezuela | 1963 |
| Thenalidine | R06 | Antihistamines for systemic use | Harm unique to the medicine | Neutropenia | 4 | 1 | 9 | USA, UK, Sweden, France, Cyprus, Australia, Finland, Norway, Venezuela | 1958 |
| Thioridazine | N05 | Psycholeptics | Harm unique to the medicine | Cardiac arrhythmias, QT prolongation | 4 | 39 | 1 | Worldwide | 2005 |
| Tolcapone | N04 | Anti-Parkinson drug | Harm unique to the medicine | Hepatotoxicity | 4 | 6 | 5 | EU, UK, Iceland, Australia, Bulgaria | 1998 |
| Tranylcypromine | N06 | Psychoanaleptics | Harm general to the chemical subgroup | Drug-drug interactions | 4 | 2 | 3 | Italy, Belgium, Venezuela | 1964 |
| Trazodone | N06 | Psychoanaleptics | Harm unique to the medicine | Carcinogenicity | 5 | 15 | 1 | Norway, | 1985 |
| Triazolam | N05 | Psycholeptics | Harm unique to the medicine | Psychiatric adverse effects | 4 | 4 | 4 | Mauritius, Norway, Brazil, UK | 1982 |
| Tryptophan | N06 | Psychoanaleptics | Harm unique to the medicine | Eosinophilia myalgia syndrome (EMS) | 4 | 3 | 5 | Switzerland, UK, Sweden, Austria, Japan | 1989 |
| Valdecoxib | M01 | Antiinflammatory and antirheumatic products | Harm general to the chemical subgroup | Cardiotoxicity, skin reactions | 2 | 1 | 3 | Canada, USA, Europe | 2005 |
| Vigabatrin | N03 | Antileptics | Harm unique to the medicine | Neurotoxicity | 5 | 18 | 1 | Norway | 1991 |
| Zopiclone | N05 | Psycholeptics | Harm unique to the medicine | Carcinogenicity | 5 | 10 | 2 | Iceland, Norway | 1986 |
